# Expression of *COLLAGEN 1* and *ELASTIN* Genes in Mitral Valvular Interstitial Cells within Microfiber Reinforced Hydrogel

**DOI:** 10.22074/cellj.2015.22

**Published:** 2015-10-07

**Authors:** Maryam Eslami, Gholamreza Javadi, Nasser Agdami, Mohammad Ali Shokrgozar

**Affiliations:** 1 Department of Biology, Science and Research Branch, Islamic Azad University, Tehran, Iran; 2 Department of Genetics,Tehran Medical Sciences Branch, Islamic Azad University, Tehran, Iran; 3 Applied Biotechnology Research Center, Tehran Medical Sciences Branch, Islamic Azad University, Tehran, Iran; 4 Department of Stem Cells and Developmental Biology, Cell Science Research Center, Royan Institute for Stem Cell Biology and Technology, ACECR, Tehran, Iran; 5 Cell Bank Division, Pasteur Institute of Iran (IPI), Tehran, Iran

**Keywords:** Tissue Engineering, Heart Valve, ELASTIN, COLLAGEN I, Real-Time PCR

## Abstract

**Objective:**

The incidence of heart valve disease is increasing worldwide and the number of heart valve replacements is expected to increase in the future. By mimicking the main tissue structures and properties of heart valve, tissue engineering offers new options for the replacements. Applying an appropriate scaffold in fabricating tissue-engineered heart valves (TEHVs) is of importance since it affects the secretion of the main extracellular matrix (ECM) components, collagen 1 and elastin, which are crucial in providing the proper mechanical properties of TEHVs.

**Materials and Methods:**

Using real-time polymerase chain reaction (PCR) in this experi- mental study, the relative expression levels of *COLLAGEN 1* and *ELASTIN* were obtained for three samples of each examined sheep mitral valvular interstitial cells (MVICs)-seeded onto electrospun poly (glycerol sebacate) (PGS)-poly (ε-caprolactone) (PCL) microfibrous, gelatin and hyaluronic acid based hydrogel-only and composite (PGS-PCL/hydrogel) scaffolds. This composite has been shown to create a synthetic three-dimensional (3D) microenvironment with appropriate mechanical and biological properties for MVICs.

**Results:**

Cell viability and metabolic activity were similar among all scaffold types. Our results showed that the level of relative expression of *COLLAGEN 1* and *ELASTIN* genes was higher in the encapsulated composite scaffolds compared to PGS-PCL-only and hydrogel-only scaffolds with the difference being statistically significant (P<0.05).

**Conclusion:**

The encapsulated composite scaffolds are more conducive to ECM secretion over the PGS-PCL-only and hydrogel-only scaffolds. This composite scaffold can serve as a model scaffold for heart valve tissue engineering.

## Introduction

Heart valve disease is a progressive disorder, with patients requiring heart valve replacement estimated to increase significantly in the future (1- 3). Until 2011, in the USA, the overall prevalence of heart valve disease had been 2.5% with a relatively wide age variation in the afflicted patients (4) and about 100,000 valve replacement surgeries were performed annually (5). There are currently two types of valves which are commonly used, namely biological valves (including autografts, xenografts and homografts) and mechanical valves which are built from synthetic biocompatible materials. Although these replacements improve survival rate of patients, none of them can fully restore native valve function; biological valves are prone to fibrosis, degeneration, calcification and immunogenic reactions while mechanical valves may cause hemorrhage and thromboembolism (6). Furthermore, existing mechanical and biological prostheses lack growth, repair and remodeling capabilities (1, 2) and they are thus not suitable for congenital heart defects and pediatric patients whose heart valves are growing (7). Tissue engineering methods can be used to overcome these limitations (8). Heart valve tissue engineering offers new possibilities to produce artificial heart valves which lack unwanted biological and mechanical properties. However, there are some problems with previously fabricated scaffolds for tissue engineered heart valves (TEHVs) (9) such as plastic deformation (10), high stiffness (11), non-anisotropic properties (12), low stability and unsuturability (13). Three common approaches of bioscaffold formation include decellularised tissues, synthetic scaffold, and preseeded composites (14), among which synthetic scaffolds are suggested to be the most suitable material for valve scaffold formation (1).

A hybrid microfibrous poly (glycerol sebacate)– poly(ε-caprolactone) (PGS-PCL) synthetic scaffold has been previously fabricated that could mimic the mechanical properties in the human valve (10). However, migration of cells out of the structure due to the high porosity of PGS-PCL and difficulties relating to the production of fully cellularized three-dimensional (3D) structures caused by the cell tendency to attach only on the surface of the scaffold are two limitations of using PGSPCL solely in TEHV (10). In our recent study, we were able to overcome these limitations by integrating PGS-PCL within a hybrid hydrogel made from methacrylated hyaluronic acid (HAMA) and methacrylated gelatin (GelMA). By creating this composite scaffold, we combined the advantages of both extracellular matrix (ECM)-mimicking hydrogels and elastomeric PGS-PCL scaffolds to imitate the cellular environment and mechanical properties of native heart valve tissue (15). All native heart valves (aortic, pulmonary, mitral and tricuspid) consist of two cell types, the valvular endothelial cells (VECs) and the valvular interstial cells (VICs). The VICs, the most prevalent cell type in native heart valves, are located throughout all layers and maintains the valvular ECM (VECM) structure of heart. The VECM has three distinct layers including the fibrosa, ventricularis and spongiosa (6, 15, 16). The predominant ECM substance in the fibrosa layer is collagen fibers, ventricularis is comprised of elastin and collagen sheets and the spongiosa, the layer between the fibrosa and the ventricularis, contains glycosaminoglycans (GAGs) and loose collagen (6, 17).

Collagen is inelastic and contributes significantly to the biomechanical strength of the heart valve tissue (18) while previous study on the role of elastin in the valve leaflet have shown that elastin maintains a specific collagen fiber configuration and return the fibers to this state, once external forces are released (19). Mol et al. (2) has shown that increasing the amount of collagen, results in improved mechanical properties of the engineered tissues.

About 34 genes are associated with collagen formation and *COL1* gene encodes the most abundant collagen of the human body. Elastin, the predominant element of elastic fibers, has a crucial role in integrity and dynamicity of tissue and paracrine signaling (20). Elastin is the main protein of ECM placed in the arterial wall and can contribute its dry weight up to 50% (6). The protein product of the *ELASTIN* gene is synthesized by vascular smooth muscle cells and secreted as a tropoelastin monomer that is soluble, non-glycosylated and highly hydrophobic. Tropoelastin is crosslinked after post-translational modifications and classified into elastin polymers. These polymers create concentric rings of elastic sheet around the medial layer of arteries. In humans, elastin is encoded by the *ELN* gene (21).

To reach an ideal TEHV with the capability of mimicking the native heart valve ECM, the relative quantity of collagen and elastin should be optimal in the TEHV. Collagen, elastin and proteoglycans account for ~60, ~10 and ~20% dry weight of the native heart valves respectively (22, 23). The normal valve has 74% type I, 24% type III and 2% type V collagen while these amounts are altered in myxomatous valves (24). Elastin disruption can produce smooth muscle sub-endothelial proliferation and thus may lead to obstructive arterial disease in mouse models (20, 25). In terms of creating a TEHV, it has been shown that the amount of VICs’ collagen production within collagen gels can be increased by adding glycosaminoglycans (26). Also, HA is an important material in fabricating TEHVs which promotes elastin production and secretion in VICs (26, 27). Changes in the quantity and structure of collagen and elastin directly alter the mechanical and functional features of TEHVs (28).

In this study, using the real-time polymerase chain reaction (PCR) technique, we compared the expression level of *COLLAGEN* and *ELASTIN* genes between VICs-seeded scaffolds constructed by either PGS-PCL or hydrogel and the newly fabricated composite scaffold consisting of both PGSPCL and hydrogel components (15).

## Materials and Methods

### Scaffold preparation

#### Synthesis of methacrylated hyaluronic acid and methacrylated gelatin

HA sodium salt (Mn: 7.52×10^5^ kDa, Lifecore Biomedical, Chaska, MN) was dissolved in deionized water and HAMA solutions were prepared in a final concentration of 1% w/v. Methacrylic anhydride (Sigma-Aldrich, St. Louis, WI) was added dropwise (2 ml per 200 ml) while the pH was kept at 8.0. The solution was placed on ice for several hours and for the pH to remain at 8, it was vital to be in 15 minutes time-steps. The solution was permitted to move around by stirring overnight in a cold room (4˚C) and was then dialyzed for two days in distilled water (dI H_2_O) with multiple solution changes using 12-14 kDa Molecular Weight Cut-Off’s (MWCO) dialysis tubes. The solutions were lyophilized for 1 week and the the final product was kept at -80˚ C.

According to the aforementioned protocol, porcine skin type A gelatin (Bloom index 300, Sigma, Madison, WI) was methacrylated (19, 29). In brief, the mixture of gelatin 10% (w/v) with Dulbecco’s phosphate-buffered saline (DPBS, Invitrogen, Grand Island, NY) was incubated at 60˚C and stirred until totally dissolved. It was vital to add methacrylic anhydride (0.8 ml/g, 8 ml) at a rate of 0.5 mL/minutes into the gelatin solution (10 g). The emulsion was agitated at 60˚C and permitted to react for 1 hour. To prevent any further reaction, warm 2x DPBS (40˚C) was added to the solution. The solution was then dialyzed for one week in distilled water using MWCO dialysis tubes (12- 14 kDa) with some solution changes. After 1 week, the freeze-drying of the solution resulted in a white porous foam that had to be preserved at -80˚C.

### Formation of poly (glycerol sebacate)-poly (ε-caprolactone) microfibrous scaffold

Through using a standard electrospinning set-up, the PGS-PCL microfibrous scaffolds were developed. Since aluminum foil is a deficient collector of stable fibers, covering a copper wire with a non-adhesive special tape with a glass slide on top was used as a collector. PGS (MW 12000, gift from Langer Laboratory, Department of Chemical Engineering, MIT) and PCL (MW70000-90000, Sigma-Aldrich, Madison, WI) were dissolved at a 2:1 ratio in anhydrous chloroform-ethanol (Sigma- Aldrich, WI, USA, 9:1 v/v) and were electrospun at 12.5 KV. The total polymer concentration was 33%. As it was hard to electrospin the highly viscous 33% w/v PCL solution, pure PCL scaffolds were electrospun by using 16% w/v polymer solution (10).

Fibers were formed at constant flow rate of 2 ml/hours by keeping the distance between the 21-gauge needle and the collector at 18 cm. Each sample was fabricated in 20 minutes. To remove any remaining solvent, the mentioned scaffolds were dried overnight in a vacuum desiccator.

### Hydrogel preparation

2.5% GelMA macromer and 0.5% HAMA macromer (as a hydrogel precursor solution) were mixed with culture media. The photoinitiator 2-hydroxy-1-(4-(hydroxyethoxy) phenyl)-2-methyl- 1-propanone (Irgacure 2959, BASF, Ludwigshafen, Germany) was added to a final concentration of 0.1% (v/w) for crosslinking the solution and it was exposed afterwards to ultraviolet (UV) light (ƛ=408 nm, 45 seconds at 2.6 mW/cm^2^). In order to control and make the composite scaffolds, the resulting hydrogel (20 μl initial solution volume, ~6 mm diameter, ~ 0.5 mm thick) was used.

### Fabrication of composite hydrogel/microfibrous poly (glycerol sebacate)–poly(ε-caprolactone) scaffolds

Composite of hydrogel/microfibrous PGS-PCL scaffolds was fabricated as previously described (15). Briefly, PGS-PCL sheets were used to create 6 mm diameter scaffolds. The PGS-PCL scaffolds were placed in contact with the precursor hydrogel solution. Soon after the whole solution was absorbed by the scaffold, UV light (ƛ =408 nm, 2.6 mW/cm^2^, 45 seconds) was used for crosslinking to get the resulting composite.

### Molecular and cellular experiments

#### Scanning electron microscopy (SEM)

To characterize the size of pores of the scaffold and the fiber morphology, scanning electron microscope (SEM) images were taken with a field emission SEM (FE-SEM, JSM 6700, JEOL, Peabody, MA). Images were taken from both surface of the hydrogel-only, the composite scaffold and the PGS-PCL-only scaffold. The samples were fixed in 2.5% gluteraldehyde, and were frozen using liquid nitrogen and then stored at -80˚C. The samples were lyophilized before sputter coating, with an iron coater of palladium and platinum. The SEM was equilibrated at 40 mA for 40 seconds.

### Mitral valvular interstitial cells (MVICs)

#### Cell culture

Sheep mitral VICs (MVICs, gift from Dr. Bischoff, Children’s Hospital, Boston, MA) with passage number between 3 and 4 were kept in endothelial basal medium (EBM-2, Lonza, Walkersville, MD) supplemented with 10% fetal bovine serum (FBS, Sigma-Aldrich, Saint Louis, MO) and 1% penicillin-streptomycin (Gibco, Langley, OK) at 37˚C and 5% CO_2_. Cell culture was done on gelatin-coated flasks where cells were passaged weekly with media changed every other day.

### Encapsulation of mitral valvular interstitial cells within hydrogels (GelMA/HAMA) and composite scaffolds

MVICs were trypsinized and re-suspended in the hydrogel precursor solution (2.5% GelMa/0.5% HAMA) having 1% (w/v) of photoinitiator (Irgacure 2959, BASF, Ludwigshafen, Germany, concentration: 6×10^6^ cells/ml). As mentioned before, both the hydrogel and composite scaffolds were made. The composites and the hydrogel precursor solution with cells were exposed to 2.6 mW/cm^2^ UV light (408 nm, 45 seconds) and were then incubated in medium (EBM2, 21 days) under standard culture conditions. In order to remove the remaining photoinitiator, the medium was changed every 30 minutes for the first two hours. The medium was then changed every other day.

### Seeding mitral valvular interstitial cells on poly (glycerol sebacate)–poly (ε-caprolactone scaffolds

PGS-PCL scaffolds were immersed in ethanol (70%, 2 hours) to be disinfected prior to seeding the cells. The scaffolds were then exposed to UV light and were washed with DPBS. The cells were seeded onto scaffolds (0.6 cm×0.6 cm) at a concentration of 2×10^4^ cells/scaffold. To seed the cells onto scaffolds, 20 μl of cell suspension was added to scaffolds in 48-well plates which were then incubated (1 hour) for the cells to attach. Every other day the medium was changed.

### Cell viability and proliferation

#### Cell viability

The cell viability of scaffolds was evaluated with the use of a live-dead assay (calcein-AM/ ethidiumhomodimer; Invitrogen, Carlsbad, CA) per manufacturer’s protocol. Cell viability studies were performed by seeding MVICs (6×10^6^ cells/ ml) with each scaffold and incubating them for 21 days. Media of the samples were changed every other day. It was fundamental to determine the viability of the cells for each type of sample particularly on days 1 and 21 of culture. To determine the percent viability, Image J software was used.

### Cell proliferation

The metabolic activity of cells on each scaffold or hydrogel from 1 to 21 days of culture was assessed by using the Alamar Blue (AB) assay in accordance with manufacturer’s protocol (Invitrogen, CA, USA). Cells were seeded onto scaffolds or were encapsulated within plain hydrogels or composite scaffolds (n=3/group). In order to avoid counting cells that had adhered to the plate surface during seeding, the cell-seeded scaffolds were transferred to new wells. While two cell-free scaffolds/ hydrogels were used as negative controls, one well which contained only medium was used to measure the background signal. AB (10% v/v) was then added to each well and samples were incubated for 2 hours at 37˚C. Afterwards, the solution from each well was transferred to wells in a 96-well plate (100 μl, n=3/group) and the fluores cence was measured at 540 nm. The 21-day measurements were normalized to the corresponding measurements from day 1.

### RNA extraction and cDNA preparation

Three samples of each VICs-seeded scaffold (PGS-PCL-only, hydrogel-only and the composite scaffolds) was examined to assess their capability to express *COLLAGEN* and *ELASTIN* genes. Total RNA was extracted from each studied sample using an RNeasy Mini Kit (Qiagen, Valencia, CA). Reverse transcription PCR (RT-PCR) was performed with a RevertAid H Minus First Strand cDNA Synthesis Kit (Thermo Scientific, PA, USA). DNAse I (Invitrogen) digestion of RNA samples (0.5 μg) was performed prior to reverse transcription.

### Real time polymerase chain reaction

Real-time PCR assay was replicated three times for each sample and the difference of the threshold cycle (Ct) values between the replicates was no more than 0.5. The average Ct was used for statistical analysis. All reactions were performed using Fast SYBR Green PCR Master Mix with the default settings on an ABI Biosystems Step One Plus Real-Time PCR Machine following: denaturation at 95˚C for 5 minutes, and 40 cycles of 95˚C for 35 seconds and 60˚C for 1 minute. Relative expression levels were determined from collected data as threshold cycle numbers. Table 1 shows the sequence of the designed primers used.

**Table 1:** Primer sequences of studied genes and the reference gene


Gene	Primer sequencing

*COL 1*	F: CCACTGGTCCCCAAATCTAA
	R: GCTTCTTTGGCAGTCTGAGG
*ElASTIN*	F: CAGCCAAATACGGTGAAACA
	R: AACACCAGGGACTCCAACAC
*GAPDH*	F: ATGCTGGTGCTGAGTACGTG
	R: GGCATTGCTGACAATCTTGA


### Data analysis

The real-time PCR technique requires that the amplification efficiencies of each studied gene to be close to 100% to allow comparative analysis of the results. The amplification efficiency (E values) obtained for all studied genes were more than 98%. Relative gene expressions were obtained using the ΔCt method of relative quantification based on the fact that the loci under study (as well as their internal control gene) are amplified with the same efficiency in each sample. The relative gene expression was calculated as 2^-ΔΔCt^, where ΔCt = C_t Target_-C_t reference_. The GAPDH gene served as an internal control.

### Statistical analysis

One-way ANOVA analysis was performed followed by post-hoc comparison in SPSS (V.15, SPSS Inc., Chicago, IL). A P value ≤0.05 was considered statistically significant. Data are reported as mean ± standard deviation (SD).

## Results

**Fig.1:**
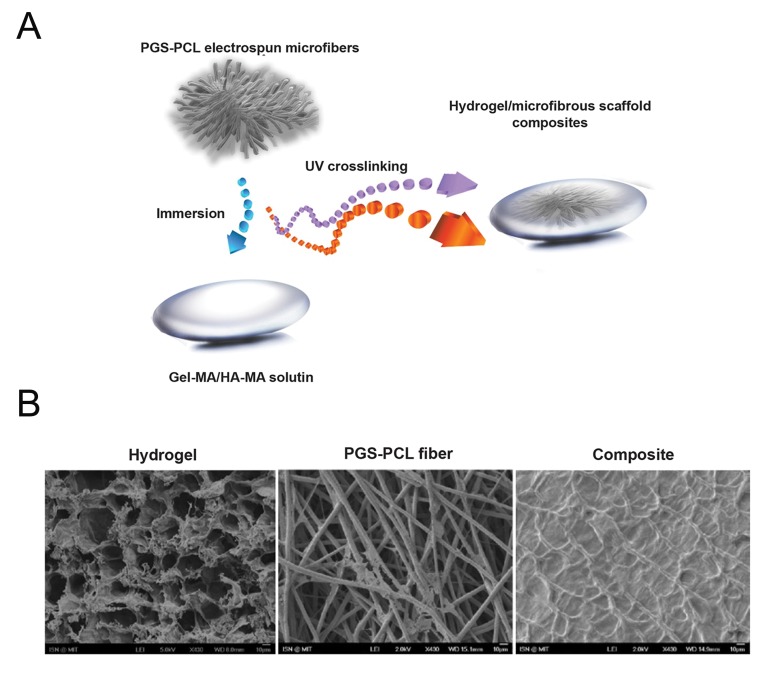
Fabrication of the fiber reinforced hydrogel (2.5% GelMA+0.5% HAMA) scaffolds. A. Schematics of the design and fabrication. Immersing the electrospun fibers inside the hydrogel precursor solution (to which cells can be added), and then crosslinking via light exposure to form a cell-laden hydrogel with electrospun fibers for reinforcement [adapted from figure 1 (15)] and B. Surface SEM images of hydrogel, PGS-PCL fiber and composite scaffolds showing the integration of the hydrogel with the electrospun microfiber. PGS; Poly (glycerol sebacate), PCL; Poly (ε-caprolactone), UV; Ultraviolet, GelMA; Methacrylated gelatin, HAMA: Methacrylated hyaluronic acid and SEM; Scanning electron microscopy.

The composite structures were synthesized using an immersion technique (15). This composite was combined by adding an ECM-like microenvironment within and around the microfibers with the addition of the hydrogel (Fig .1A). Because of the utilization of hydrophobic polymers, fiber/hydrogel composite constructs were prepared by simultaneous electrospinning of the microfibers and electrospraying of the hydrogel (28). The advantage of this method was due to the ability of the PGS-PCL microfibers in absorbing the hydrogel precursor solution. The composite was manufactured after electrospinning and cells were directly encapsulated within the composite during the hydrogel crosslinking step. The PGS component of the microfibers was necessary to simplify this complete interface because the GelMA/HAMA gel solution was unable to penetrate the PCL-only fibers (15). The presence of PGS renders PGSPCL microfibrous scaffolds more hydrophilic thus enabling their further modification (10). Additionally, the gel solution was absorbed more quickly when the scaffold was submerged in the gel solution compared to directly adding it to the scaffold structure (Fig .1A). SEM images of the composites show that the scaffold microfibers were encapsulated within a layer of hydrogel (Fig .1B).

**Fig.2:**
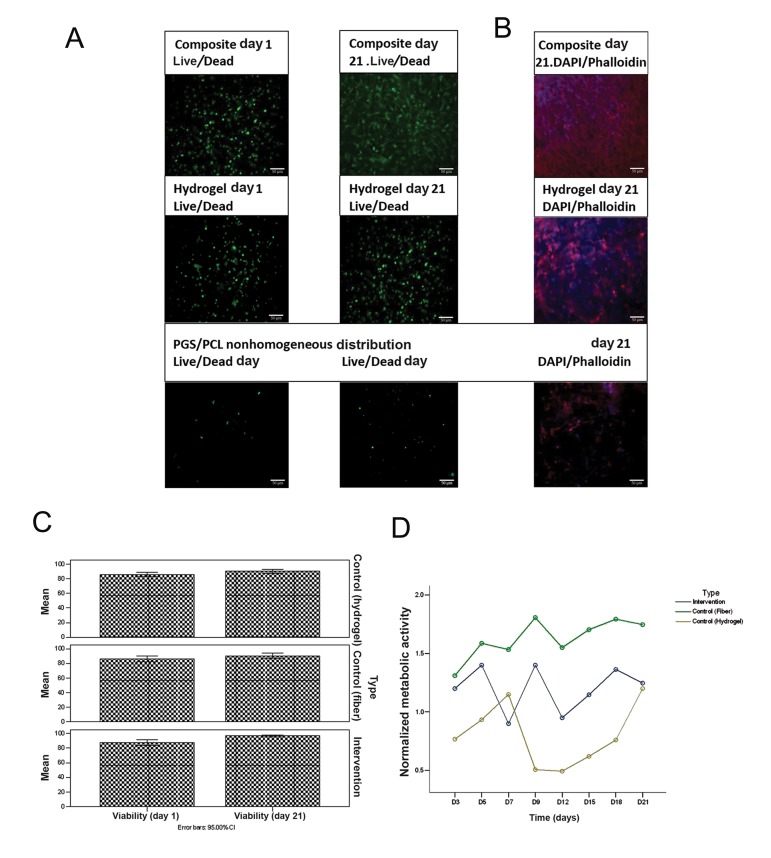
The behavior of cells encapsulated in composite scaffolds compared with cells seeded on PGS-PCL fibers and/or encapsulated in GelMA/HAMA hydrogel alone. A. Viability of the encapsulated or seeded cells as observed through Live/Dead staining of the MVICs on days 1 and 21, B. DAPI/phalloidin staining on day 21 showing cell spreading and distribution, C. Quantification of the Live/Dead images by Image J (n=3) and D. Metabolic activity determined using Alamar Blue (n=3, P<0.05). PGS; Poly (glycerol sebacate), PCL; Poly (ε-caprolactone), GelMA; Methacrylated gelatin, HAMa; Methacrylated hyaluronic acid and DAPI; 4, 6-diamidino-2-phenylindole.

The initial encapsulation process maintained a high level of cell viability (≥90%). At day 21, the viability of MVICs was still above 90% for all three scaffolds (Table 2), accompanied by a significant increase in the cell number (Fig .2A). The metabolic activity of seeded cells was used as an indirect method to estimate cell number. The cells within the composite scaffolds showed higher metabolic activity compared to those on the scaffold only or within the hydrogel samples at all time points (Table 3). The metabolic activity showed distinct peaks at different time points for the samples. This may be caused by cells getting a quiescent status when a defined cellular density is reached (Fig . 2B).

**Table 2:** 


Type		Viability (day 1)	Viability (day 2)

Copmosite	Mean	87.67	96.67
	Std. deviation	1.528	0.577
Control (fiber)	Mean	86.33	90.33
	Std. deviation	1.528	1.528
Control (hydrogel)	Mean	86.00	90.00
	Std. deviation	1.000	1.000


MVICs; Mitral valvular interstitial cells.

**Table 3:** Metabolic activity determined using Alamar Blue (n=3)


Type		NMA (day 3)	NMA (day 5)	NMA (day 7)	NMA (day 9)	NMA (day 12)	NMA (day 15)	NMA (day 18)	NMA (day 21)

Composite	Mean	1.200	1.400	0.900	1.400	0.950	1.147	1.363	1.247
	Std. deviation	0.0500	0.0500	0.0500	0.0500	0.0500	0.0252	0.0709	0.0351
Control (fiber)	Mean	1.310	1.587	1.533	1.807	1.550	1.703	1.793	1.747
	Std. deviation	0.0854	0.0709	0.0351	0.0702	0.0300	0.611	0.0902	0.0252
Control (hydrogel)	Mean	0.767	0.933	1.150	0.507	0.493	0.620	0.760	1.200
	Std. deviation	0.0208	0.0289	0.0400	0.0321	0.0306	0.0200	0.5973	0.2000


NMA; Normalized metabolic activity.

**Fig.3:**
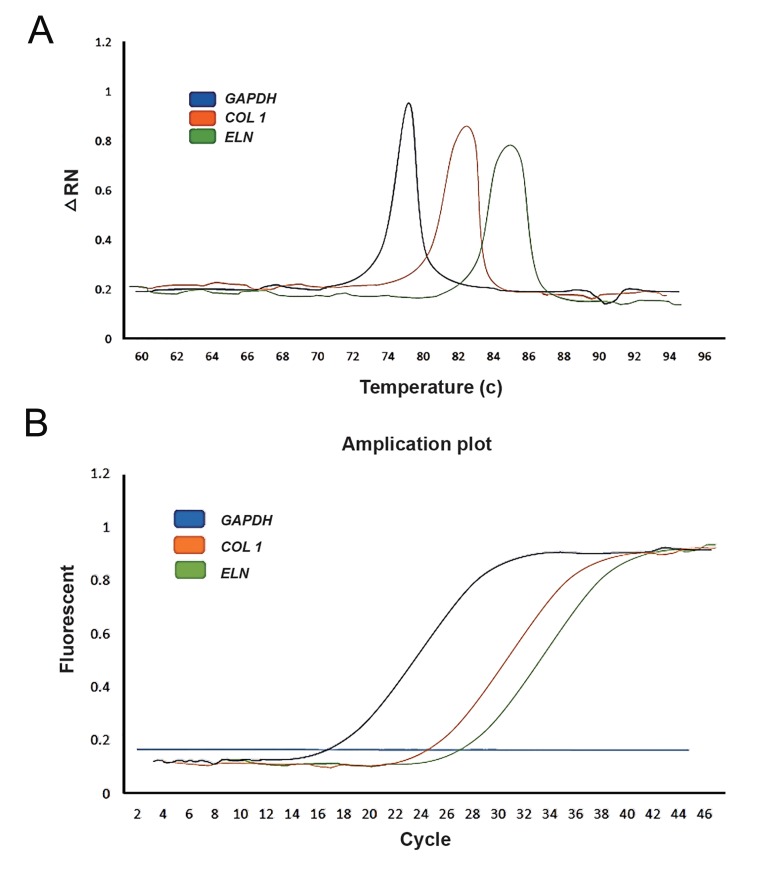
Real-time PCR assay of COLLAGEN 1, ELASTIN and GAPDH gene expression in the intervention sample. A. Melt curve analysis of three studied genes (Blue; GAPDH, Orange; COLLAGEN 1 and Green; ELASTIN) and B. Amplification plot of the three genes (Blue; GAPDH, Orange; COLLAGEN 1 and Green; ELASTIN).

**Fig.4:**
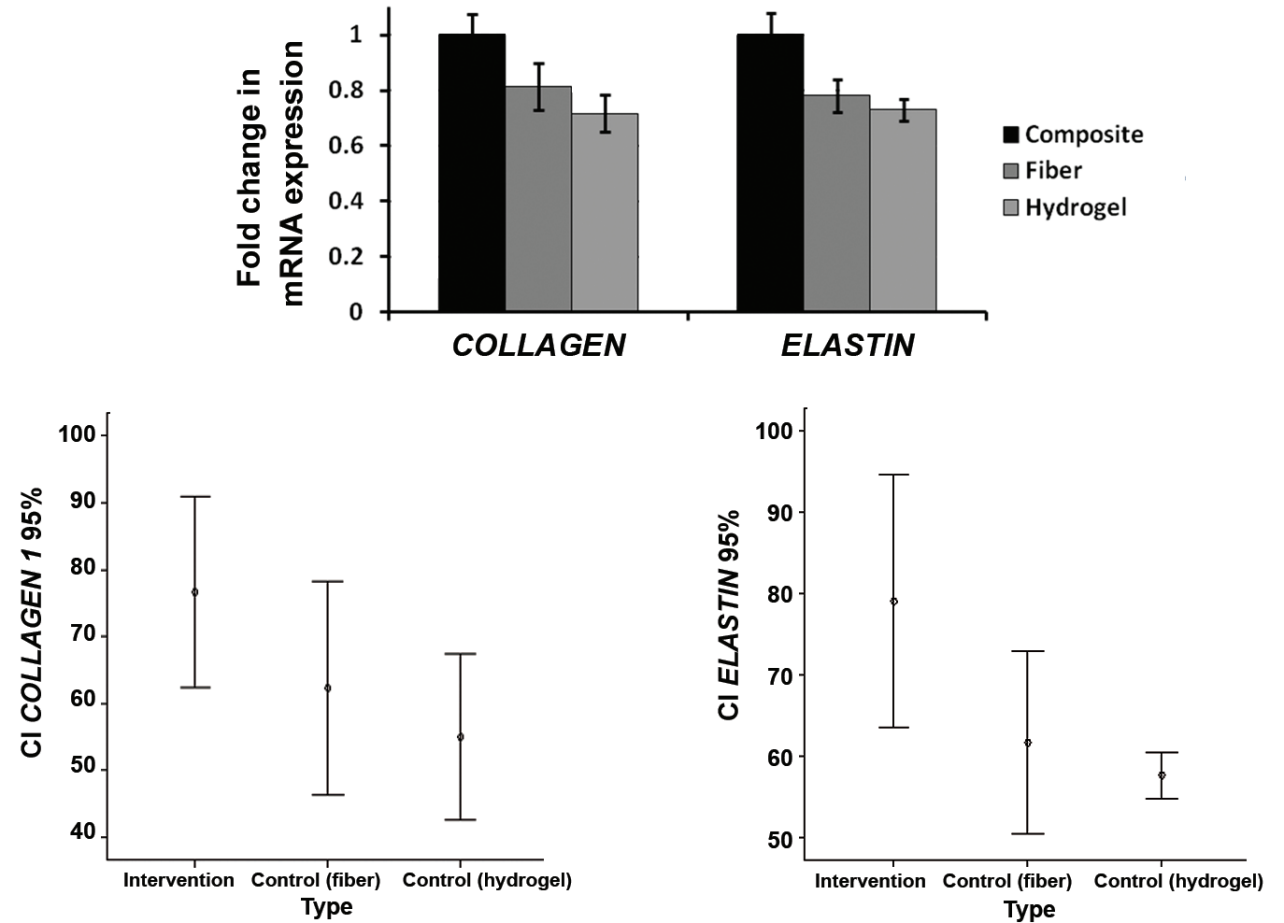
Level of COLLAGEN 1 and ELASTIN genes expression in three studied sample types (intervention, fiber and hydrogel). Level of COLLAGEN 1 and ELASTIN genes expression are highest in intervention case in comparison with PGS-PCL fiber-only and Hydrogel-only cases (n=3, P=0.010). PGS; Poly (glycerol sebacate) and PCL; Poly (ε-caprolactone).

Our statistical analysis showed that the level of *COLLAGEN 1* gene expression was higher in the VICs-seeded composite scaffold compared with PGS-PCL-only and hydrogel-only scaffolds (Fig .3) and the difference was statistically significant (P=0.010). The most and the least *COLLAGEN 1* gene expression percent were related to the composite (76.67 ± 5.8%) and hydrogel-only (55 ± 5%) scaffolds respectively. The difference of *ELASTIN* gene expression level between the composite, PGS-PCL-only and hydrogel-only scaffolds was also statistically highly significant (P=0.003) with the highest level in the composite scaffold (79 ± 6.2%) and the lowest level in the hydrogel-only scaffold (57.67 ± 3%). The *COLLAGEN 1* gene expression of the hydrogel-only and PGS-PCL-only scaffolds was decreased 0.21 and 0.19 fold in comparison to composite scaffold respectively. The corresponding values for *ELASTIN* were 0.27 and 0.22 fold respectively. The *COLLAGEN-ELASTIN* genes expression ratio in the composite scaffold was 1 (Fig .4).

## Discussion

VICs have a heterogeneous phenotype resulting from the ECM layered structure with fibrous, collagen- rich and glycosaminoglycan-rich regions. This special structure is necessary for heart valves to tolerate significant mechanical forces such as stretch and flexure during diastole and systole respectively (16). Thus, choosing appropriate biomaterials and synthetic scaffolds is a crucial step in fabricating TEHVs especially because they affect the quantity and architecture of ECM components which can directly alter the mechanical, elastic and tensile strength of TEHVs (30).

The main proteins secreted by VICs are collagen (types I, III and IV), laminin and fibronectin (31) among which collagen has a considerable role in maintaining tissue integrity and providing mechanical strength (32). Ramamurthi and Vesely (26) have shown that by adding glycosaminoglycans, the amount of VICs’ collagen production in collagen gels can be increased. Sant et al. (33) investigated the ability of VICs seeded on fibrous PGS-PCL scaffold to secrete fibrous proteins, specifically collagen type I. Recently, Masoumi et al. (9) demonstrated that scaffolds with (2:1) PGSPCL polymer ratios have the highest level of *COLLAGEN* expression.

Similarly, in the current study, we assessed the ability of collagen type 1 secretion by VICs seeded on PGS-PCL-only scaffold and obtained the level of *COLLAGEN* gene expression using real-time PCR. The necessary elastic properties in native heart valves are obtained by elastin, another major ECM component. Sant et al. (33) also evaluated elastin secretion by VICs on PGS-PCL fibrous scaffold and did not find any elastin secretion by VICs in their study. Previous studies have shown that HA substrates promote the production of elastin by smooth muscle cells (26, 27). Masters et al. (34) in a study on VICs cultured on tissue culture polystyrene, reported an increase in elastin production and a decrease in collagen production in response to the delivery of HA oligosaccharides. In the current study, the level of *ELASTIN* gene expression was obtained in the hydrogel-only scaffold which consisted of both HAMA (promoting elastin secretion) and GelMA (promoting cell spreading) (22). We also investigated the expression level of *COLLAGEN*E in the hydrogelonly scaffold. We further assessed the quantity of *COLLAGEN* and *ELASTIN* genes expression in our recently fabricated VIC-seeded composite scaffold consisting of both PGS-PCL (with its collagenous layers providing mechanical strength) and the hydrogel component (GelMA/HAMA; providing a 3D glycosaminoglycan rich ECM-like microenvironment). We then compared the obtained quantities with those from PGS-PCL-only and hydrogelonly scaffolds.

## Conclusion

Our results demonstrate that the expression level of *COLLAGEN* and *ELASTIN* genes was statistically significantly higher in the composite scaffold compared with those of PGS-PCL-only scaffold and hydrogel-only scaffold. The optimal expression levels of *ELASTIN* and *COLLAGEN* genes in the VIC-seeded composite scaffold demonstrate the superiority of this composite compared with the PGS-PCL-only and hydrogel-only scaffolds. This composite scaffold can serve as a model scaffold for heart valve tissue engineering which can provide the necessary mechanical, elastic and tensile strength. Furthermore, this composite scaffold has the capability to grow, be repaired and remodeled and may thus be suitable for congenital heart defects.
